# Exploring the relationship between mental health issues and academic performance of undergraduate students in a Ghanaian tertiary institution: a cross-sectional study

**DOI:** 10.1007/s44192-025-00130-8

**Published:** 2025-01-21

**Authors:** Andrew Nketsia Arthur, Joanne Fraikue, Bijoux Adu-Amankwah, Richard Ofori, Dorcas Sekyi, Abena Afrakomah Boateng, Elsie Asamoah, Seth Christopher Yaw Appiah

**Affiliations:** Department of Sociology and Social Work, Faculty of Social Sciences, Kwame Nkrumah University of Science and Technology, Kumasi, Ghana

**Keywords:** Depression, Anxiety, Stress, Cumulative Weighted Average (CWA), Academic self-efficacy

## Abstract

**Background:**

Mental health associations with students’ academic outcomes are critical for students’ well-being and excellent performance, particularly among tertiary students in their educational trajectory. This study investigated the relationship between mental health incidence and academic performance among university students in a public university in Ghana. Additionally, we study students’ level of mental health awareness.

**Methods:**

The study adopted a quantitative study with an analytical cross-sectional design. Through the multistage sampling technique, structured questionnaires were administered to 384 undergraduate students at Kwame Nkrumah University of Science and Technology (KNUST). Data management and analysis were performed using SPSS v.27. Descriptive data are presented in tables and reported as frequencies. Inferential analysis was conducted using a multilevel logistic regression model and a p-value of 0.05 was considered to indicate statistical significance.

**Results:**

Severe depression (p = 0.016, ϰ^2^ = 0.784) was significantly associated with students’ academic performance. Severe anxiety (p = 0.020, ϰ^2^ = 11.700), gender (p = 0.014, ϰ^2^ = 5.999) and self-efficacy (p = 0.015, ϰ^2^ = 5.939), were found to have a statistically significant association with academic performance (p < 0.05). According the multilevel analysis, females experiencing mental health had reduced likelihood of achieving high academic performance compared to male students (AOR = 0.343, 95% CI 0.144–0.813, p = 0.015). Severely depressed students (AOR = 0.264, 95% CI 0.084-0.830, p = 0.23) are significantly less likely to increase their Cumulative Weighted Average (CWA). Self-efficacious students (AOR = 3.325, 95% CI 1.421-7.784, p = 0.06) were three times more likely to increase their CWA.

**Conclusion:**

Severe depression causes a reduction in students’ academic performance, while high academic self-efficacy among students serves as a protective factor by facilitating an increase in students’ CWA when they encounter mental health issues.

## Background

Mental health difficulties and enveloping conditions such as depression, anxiety, and stress have garnered increased global attention among educationists, health professionals and policy stakeholders with an elevated level among university students [1–3]. The transition from childhood to maturity (aged 18–29 years) is a developmental period characterized by shifts in autonomy (e.g., moving out of the house, being required to plan one’s education), relational insecurity, and changes in anticipated capacity [4–6]. Students encounter educational, social and financial challenges, the majority of which increase their stress levels as they climb the academic ladder [7, 8].

It has further been reported that one out of three university students encounter mental health issues throughout their studies with a comparable percentage quitting tertiary schooling without graduating with a degree [9]. Students in higher educational institutions experience greater stress than those in lower institutions [10]. At Jizan University in Saudi Arabia, [11] reported that stress was prevalent among medical students, with females reporting higher levels of stress (77%) than males (64%). Up to one-third of students could experience moderate to severe depression at any given moment, a proportion that may be greater than that of the general population [12]. In a recent study by [5], which included students from 19 institutions in eight different countries (N = 13,984), generalized anxiety disorders and depression were identified as the most prevalent mental health conditions faced by students.

Compared to their male colleagues, female students in their first year of study are more likely to experience mental health issues [13]. Studies have indicated that students with depressive symptoms seldom perform well academically [14, 15].

The primary academic pressures for Ghanaian undergraduate students have been reported to include heavy workloads, lack of resources, inadequate enthusiasm, unsatisfactory performance, and uncertainty regarding employment after graduation [16]. Additionally, academic achievement is impacted by several health-related factors, such as depressive symptoms, low self-esteem, and poor mood [17], general mental health issues [18], and emotional intelligence [19].

The prevalence of depression among tertiary students in Ghana is approximately 39.2% [20]. Female students have worse mental health than male students and are more likely to experience depression while attending university [21]. However, the specific reasons for this phenomenon have not been explored in the extant literature.

In a study conducted by [22] to identify the factors that contribute to anxiety and depression in female medical students at King Abdulaziz University, 34.9% of the students reported having anxiety. Constrained academic schedules and poor academic achievement in the six months before the research were established as the primary causes of anxiety.

Despite considerable studies that have established that depression, anxiety and stress are fairly common among undergraduates, there has been a growing concern about how they affect academic performance [23, 24]. Among the few reported empirical studies that have been conducted in Ghana despite the scenarios of occurrence among students, the focus has been on pupils in basic schools [25, 26], with little attention given to tertiary institutions where higher levels of stress are perceived or anticipated to exist due to the heavy workload, pressure to perform and lack of flexibility. The mental wellness of students in several Western countries has been the subject of extensive research [24, 27–29], with very few extensive studies been conducted in the global south. More so, the findings may not be generalizable because culture and context can significantly influence how depression, anxiety, and stress are perceived and experienced. This highlights the need for further research on how depression, anxiety and stress factors influence academic performance in the Ghanaian setting. To provide support for the development of essential policy intervention services for those experiencing stress, depression and anxiety in the university environment.

## Methods

### Study design and setting

A cross-sectional design was adopted for this study as it allowed the researcher to explore key characteristics and test for associations between variables of interest. The study took place at the Kwame Nkrumah University of Science and Technology, Kumasi-Ghana. The tertiary institution is among the six publicly owned universities in Ghana.

### Population and sampling

The study's target population consists of KNUST undergraduate students. [30] Statistical formula was used to estimate a sample of 384 students out of the 85000 undergraduate students who were recorded during the 2022/2023 academic year. The sample size for the study, as determined with the formula was 384. The sample was increased to 403 to compensate for questionnaire incompleteness and dropouts. Adopting a multistage sampling technique, the six colleges were purposefully selected from the university based on their representation of diverse student populations at the first stage. This comprised of College of Science, College of Engineering, College of Art and Built Environment, College of Humanities and Social Sciences, College of Agriculture and Natural Resources, and College of Health Sciences. In the second stage, each selected college was stratified into three groups: First year, Continuing year and final year. A simple random sampling technique was used to select the required number of students from each year group within each college. This sampling technique is useful because it creates a sample that truly represents the population [31].

Students who are enrolled as undergraduate students aged 18 years and older and willing to particpate were eligible for the study. Exclusion criteria included students who are unable to provide legal informed consent, and non-regular students.

### Sample size calculation

Krejcie and Morgan (1970) formula $$\left( {s = \begin{array}{*{20}c} {{\rm X}^{2} NP\left( {1 - P} \right)} \\ { d^{2} \left( {N - 1} \right) + {\rm X}^{2} P\left( {1 - P} \right)} \\ \end{array} } \right)$$ was used to determine the sample size of this study. Where *‘s’* is the sample size, *‘X*^2’^ is the chi-square for 1 degree of freedom at the desired level (3.841), ‘*N’* is the population size (85,000), ‘*P’* is the population proportion (0.50) as this will give the maximum sample size and ‘*d’* is the margin of error expressed as a proportion (0.0499). Thus, the sample size *s* = 384. However, a 5% contingency rate was applied to account for incomplete questionnaires. Hence, the final sample size was increased to 403.

### Data collection

A closed-ended questionnaire was used to obtain data from respondents with the aid of the GIC Collect, KoboCollect, and ODK Collect software applications. This instrument saves time and is cost-effective.

### Pilot testing

A pilot study was conducted with a small sample of 30 students at the Faculty of Social Sciences, College of Humanities and Social Sciences lecture block to assess the clarity and feasibility of the questionnaire.

### Questionnaire development and validation

The questionnaire was developed based on a review of relevant literature and expert examination. The instrument included both demographic questions and validated scales to assess mental health issues including the DASS-21 and PSS-4 as well as academic self-efficacy (GASE-3) scale item.

### Measurement

The level of students’ mental health issues was assessed by using the Depression Anxiety Stress Scale (DASS-21) to measure depression and anxiety, Perceived Stress Scale (PSS-4) to measure perceived stress among students, and the General Academic Self-efficacy (GASE-3: Nielsen) designed to measure academic self-efficacy. A total of 14 items were selected from the DASS-21; consisting of depression (7 items) and anxiety (7 items). Depression subscale assesses hopelessness, devaluing one’s own life, drive, disinterest, and negative thinking. The anxiety subscale evaluated circumstantial anxiety, restlessness, uneasiness, and subjective experience of worry. The PSS-4 was used to assess student’s subjective perception of stress.

Scores on the DASS-21 were multiplied by 2 to calculate the final score. Scores on the depression subscale range from zero (0) to forty-two (42). Higher scores represent higher levels of depression while lower scores represent lower levels of depression. Normal depression ranges from zero (0) to nine (9), Mild depression covers scores from ten (10) to thirteen (13), a score of fourteen (14) to twenty (20) was rated as Moderate depression, Severe depression was marked from a score of twenty-one (21) to twenty-seven (27) and Very Severe depression ranges from twenty-eight (28) to forty-two (42). Scores on the anxiety subscale range from zero (0) to forty-two (42). Higher scores represent higher levels of anxiety while lower scores represent lower levels of anxiety. A score from zero (0) to seven (7) depicted Normal anxiety, Mild anxiety scores were marked from eight (8) to nine (9), Moderate anxiety ranges from ten (10) to fourteen (14), score of fifteen (15) through nineteen (19) indicated Severe anxiety and Very Severe anxiety was from twenty (20) to forty-two (42).

Students rated each item on a 5-point Likert scale; from 0 (Never) to 4 (Very often), depending on the degree they have experienced each event, feeling, or situation over the past month. However, two of the PSS-4 positive items are reverse scored (Item 2 and Item 3); that is, 0** = **4, 1 = 3, 2 = 2, 3 = 1, 4 = 0. PSS-4 individual scores range from 0 to 16 with higher scores indicating higher perceived stress. Scores ranging from 0–5 would be considered low-stress, 6–10 moderate and 11–16 considered as high perceived stress. Lower scores indicate lower perceived stress levels, as higher scores suggest higher perceived stress.

The GASE-3 consists of only three items, that assesses academic self-efficacy beliefs. The five-item self-report scale measures academic self-efficacy on a five-point Likert scale ranging from 1 (strongly disagree) to 5 (strongly agree). Low Academic Self-Efficacy ranges from zero (0) to five (5). A score range from six (6) to ten (10) was rated as Moderate Academic Self-Efficacy and scores of eleven (11) through fifteen (15) denotes High Academic Self-Efficacy.

The internal consistency of scale items was assessed to ensure reliability using Cronbach’s alpha. Cronbach’s alpha coefficients for depression and anxiety subscale (DASS-21), PSS-4 and GASE-3 were (*a*) = 0.79, (*a*) = 0.73, and (*a*) = 0.84, respectively, indicating good internal consistency.

Students’ academic performance was determined based on their Cumulative Weighted Average (CWA). Participants provided information about their most recent CWA. CWA is a grading system used in KNUST. The system is centered on a student’s weighted average marks, with the final grade being derived and expressed as a percentage, between 0 and 100.

### Ethical consideration

Consent was obtained from the respondents before they participated in the study. Furthermore, the respondents were assured of confidentiality by ensuring anonymity thus, personally identifiable student data such as names, phone numbers, email addresses, and physical characteristics, were not collected. Questions related to the study that were raised by respondents were duly addressed.

### Data analysis

Inferential and descriptive data analysis was performed using the IBM Statistical Package for Social Sciences (IBM SPSS) version 27. Both statistics are presented in frequency tables and descriptive summaries were utilized to provide a comprehensive overview of the respondents’ sociodemographic characteristics. A Pearson chi-square test was conducted to examine the associations between mental health variables and academic performance. Both binary and multilevel logistic analyses were performed to examine the independent predictors of academic performance. As a result of the unique predictive ability of the outcome variable of interest, the variables of age, year of students, mental health awareness and stress were also added to the multilevel logistics analysis to explore their predictive effect on the outcome variable of interest. Predicting variables, including depression and anxiety, were recalibrated such that normal, mild, and moderate depression or anxiety were coded as normal. Severe and very severe depression or anxiety were coded as severe depression or anxiety, respectively. Stress was recategorized into a dichotomous variable and measured as low stress or high stress. Self-efficacy was reclassified from low and moderate self-efficacy to low self-efficacy while high self-efficacy remained unchanged. The main outcome variable was academic performance (CWA). This was recategorized from the preliminary form: 39.99 and below was termed “Failure to Graduate”, 40.00 to 49.99 (Pass) and 50.00 to 59.99 (Second Class lower) were recoded as “Low academic performance”, and 60.00 to 69.99 (Second Class Upper) and 70.00 and above (First Class) were considered “High academic performance”. In the logistic regression table, the crude (COR) and adjusted (AOR) odds ratios along with their respective confidence intervals (CIs) are presented. Variables found to be significant at p < 0.05 were entered into the binary logistic regression, and those significant at the crude level were entered into the multilevel logistic regression. The crude odds ratio (COR) and adjusted odds ratio (COR) were approximated to confidence intervals of 95%.

## Results

### Sociodemographic

Over half of the students were females (52.9%), 292 (76.0%) were within the 21–24 age group and were in their final year (52.1%). The majority of the students (355, 92.4%) performed well in their academics. Table 1 presents the obtained sociodemographic data.

**Table 1:** Sociodemographics of the students

Variable	Frequency (%)
Gender	
- Female	203 (52.9)
- Male	181 (47.1)
Age	
- 17—20	67 (17.4)
- 21 – 24	292 (76.0)
- 25 and above	25 (6.5)
Year of Study	
- First Year	62 (16.5)
- Continuing Year	122 (31.8)
- Final Year	200 (52.1)
Cumulated Weighted Average (CWA)	
- Low	29 (7.6)
- High	355 (92.4)

Table 2 displays the levels of the students’ mental health issues. The 14-item sub-scale of the Depression, Anxiety, and Stress Scale (DASS-21) was used to assess depression and anxiety levels, and the responses are presented as frequencies (percentages). Students’ depression levels were recoded as normal depression or severe depression*.* Anxiety level was rated as either normal anxiety or severe anxiety*.* The stress levels were recategorized as low stress or high stress. Academic self-efficacy levels were categorized into low self-efficacy and high self-efficacy.

**Table 2:** Mental health experiences among university students

Variable	Frequency (%)
Depression	
- Normal Depression	305 (79.4%)
- Severe Depression	79 (20.6%)
Anxiety	
- Normal Anxiety	250 (65.1%)
- Severe Anxiety	134 (34.9%)
Stress	
- Low Stress	361 (84.0%)
- High Stress	23 (6.0%)
Mental Health Awareness	
- Low Awareness	241 (62.8%)
- High Awareness	143 (37.2%)
Academic Self-Efficacy	
- Low Self-Efficacy	121 (31.5%)
- High Self-Efficacy	263 (68.5%)

A minority of the students, 39 (10.2%), reported severe depression, and almost two-thirds of the students, 250 (65.1%), said that they experienced severe anxiety. A total of 361 (84.0%) students who responded to this question experienced low stress. High academic self-efficacy was self-reported among 263 (68.5%) students. The students were assessed for their overall level of mental health awareness and more than 200 of them (62.8%) experienced low mental health awareness.

### Associations between mental health variables and academic performance

The association between mental health and academic achievement was investigated by employing a chi-square test with a p-value of 0.05 (Table 3). Depression (p = 0.016, ϰ^2^ = 0.784), anxiety (p = 0.020, ϰ^2^ = 11.700), gender (p = 0.014, ϰ^2^ = 5.999), and self-efficacy (p = 0.015, ϰ^2^ = 5.939) were found to be significantly associated with the academic performance of students (p < 0.05).

**Table 3:** Mental health association with academic performance

Variable	Academic performance	Chi^2^ value X^2^	P-value
	High performance (%)		
Gender			
- Male	161 (89.0%)	5.999^a^	0.014*
- Female	194 (95.6%)		
Age			
- 20 and below	61 (91.0%)	1.104^a^	0.576
- 21 – 24	272 (93.2%)		
- 25 and above	22 (88.0%)		
Academic Year			
- First year	52 (83.9%)	8.404^a^	0.015*
- Continuing year	113 (92.6%)		
- Final year	190 (95.0%)		
Awareness			
- Low Awareness	133 (93.0%)	0.102^a^	0.749
- High Awareness	222 (92.1%)		
Depression			
- Normal Depression	287 (94.1%)	0.784^a^	0.016*
- Severe Depression	68 (86.1%)		
Anxiety			
- Normal Anxiety	234 (93.6%)	11.700^a^	0.020*
- Severe Anxiety	121 (90.3%)		
Stress			
- Normal Stress	335 (92.8%)	1.057^a^	0.304
- High Stress	20 (87.0%)		
Self-Efficacy			
- Low Self-Efficacy	106 (87.6%)	5.939^a^	0.015*
- High Self-Efficacy	249 (94.7%)		

*Results with significant p-values (p<0.05)

^a^The chi square value at which the variables are considered statistically significantly associated at p<0.05

### Predictors of academic performance

The predictors of academic performance were examined using a multilevel regression model (Table 4). At the adjusted level, females were significantly less likely to report greater academic achievement (AOR = 0.343, 95% CI 0.144–0.813, p = 0.015) than males were. Students who reported severe depression were significantly less likely to achieve greater academic performance (AOR = 0.264, 95% CI 0.084–0.830, p = 0.023) than those whose depression level was rated as normal. Students possessing high academic self-efficacy were three times more likely to attain high academic achievement (AOR = 3.325, 95% CI 1.421–7.784, p = 0.006).

**Table 4:** Predictors of academic performance

Variable	Academic Performance	COR (95% C.I)	P-value	AOR (95% C.I)	P-value
	High Performance				
Gender					
- Male	161 (89.0%)	1		1	
- Female	194 (95.6%)	0.373 (0.165–0.843)	0.018*	0.343 (0.144–0.813)	0.015*
Age					
- 25 and above	22 (88.0%)	1		1	
- 20 and below	61 (91.0%)	1.386 (0.319–6.024)	0.663	3.851 (0.723–20.499)	0.114
- 21 – 24 years	272 (93.2%)	1.855 (.511–6.730)	0.348	1.745 (0.445–6.840)	0.424
Academic Year					
- First year	52 (83.9%)	1		1	
- Continuing year	113 (92.6%)	2.415 (0.926–6.927)	0.071	3.640 (1.146–11.563)	0.028*
- Final year	190 (95.0%)	3.654 (1.444–9.248)	0.006*	6.158 (1.891–20.051)	0.003*
Awareness					
- Low Awareness	133 (93.0%)	1		1	
- High Awareness	222 (92.1%)	1.138 (0.514–2.521)	0.750	1.133 (0.493–2.605)	0.768
Depression					
- Normal depression	287 (94.1%)	1		1	
- Severe depression	68 (86.1%)	0.388 (0.175–0.859)	0.020*	0.264 (0.084–0.830)	0.023*
Anxiety					
- Normal Anxiety	234 (93.6%)	1		1	
- Severe Anxiety	121 (90.3%)	0.636 (0.296–1.366)	0.246	1.956 (0.636–6.015)	0.242
Stress					
- Normal stress	335 (92.8%)	1		1	
- High stress	20 (87.0%)	0.517 (0.144–1.856)	0.312	.481 (0.120–1.918)	0.300
Self-efficacy					
- Low Self-Efficacy	106 (87.6%)	1		1	
- High Self-Efficacy	249 (94.7%)	2.517 (1.174–5.398)	0.018*	3.325 (1.421–7.784)	0.006*

COR connotes the crude Odds Ratio, AOR means adjusted Odds Ratio, CI is the Confidence Interval. *Significant association at p ≤ *0.05*

Continuing students were three times more likely to achieve greater academic performance than first-year students (AOR = 3.64, 95% CI 1.146–11.563, p = 0.028). Final-year students are half a dozen times more likely than freshers to perform better in their academics (AOR = 6.15, 95% CI 1.891–20.051, p = 0.003).

However, students’ awareness level, age, severe anxiety and stress were found to have no statistically significant relationship with high academic performance. Demonstrating a significant association between gender, depression, anxiety, year of study, and self-efficacy with academic performance.

## Discussion

This cross-sectional study explored the relationship between mental health issues and academic performance among undergraduate students in a public tertiary institution. Severe depression (86%) was noted to reduce students’ academic performance. Severely depressed students had a reduced likelihood of achieving high academic performance as compared to students who had experienced normal depression (AOR = 0.264, 95% CI 0.084–0.830, p = 0.023). This may potentially explain the inverse influence of severe depression on academic performance. As the severity of depression increasess, students may struggle more with the physical, cognitive and emotional demands of their academic responsibilities. Severe depression can lead to students’ reduced concentration, low productivity, disruption in learning and decreased optimism, which collectively hinder a student’s ability to perform well academically.

This finding is consistent with earlier studies among university students at Western Michigan University, where [32] reported that depression was associated with a 0.49 point, or half-letter score decline in the student Grade Point Average (GPA). Similar to the above finding, among African students from South Africa, [33] reported that the likelihood of progression delay increased with the severity of depression symptoms among undergraduate students.

It is possible that weighted academic schedules, examinations, emotional issues, little to no psychological assistance to students on campus, and the conjecture of unavailable employment opportunities immediately after graduation in Ghana caused severe depression.

Our findings rather do not show similar expressions and differ in finding compared with a study conducted in Pakistan by [34] where no relationship was established between students’ mental health issues and academic achievement. Although there was no association between mental health challenges and students’ outcomes as corroborated by [35] research in North-East Ibadan, which focused on high school students, our findings posit otherwise. In contrast, our results coming from lower and middle-income settings among tertiary students affirm the near absence of supportive mental health counselling structures to support students constrained by depression, anxiety and stress in higher institutions of learning.

According to our multiple regression analysis, anxiety was not significantly associated with academic performance. This finding is not supported by previous studies in Kuwait [36] and the USA [37], where anxiety was found to be significantly associated with academic performance and impact same. This rather contradictory result may be that anxiety can be a propelling element of high academic performance, as it drives students to invest tremendous effort in their academics, an observation that has been made by [38] in their study of Indonesian state university students, where severe academic anxiety can have an impact on the efficacy of a student’s daily work.

Our findings demonstrate that stress has no significant association with students' academic performance. This finding is not supported by earlier studies in Pakistan [39], where stress was found to have a moderate negative correlation with academic performance. In Peru, evidence from previous works [40] established that stress is used as a form of booster to achieve educational goals. This relationship may be partly explained by the fact that stress can be considered an advantage in disguising students playing a positive or manageable role that can motivate them to improve their performance and achieve academic success [41].

Further analysis of the data revealed gender differences in academic performance among individuals with mental health issues. As Table 4 shows, there was a significant difference (AOR = 0.343, 95% CI 0.144–0.813, p = 0.015) between males and females. The results showed that male students had higher odds of performing better in their academics than their female colleagues when experiencing mental health issues. However, these results differ from those of some published studies [2, 28]. This result is somewhat counterintuitive.

Final-year students were found to be six times more likely (AOR = 6.15, 95% CI 1.891–20.051, p = 0.003) than freshers to perform better in their academics. Contextually, first-year students have moved away from senior high school to tertiary education and are yet to adapt to the learning and studying process at the university. Final-year students are mostly grounded, and those who have set higher academic targets are more concerned about achieving them. There is also a perfect understanding of how their results are and have seen their mistakes over the previous semesters and remain experienced in their ability to recognize stressors, develop strategies to intervene in depression and anxiety symptoms and are more experienced in managing academic challenges.

Compared with first-year students, continuing students were three times more likely to achieve high academic performance (AOR = 3.64, 95% CI 1.146–11.563, p = 0.028). Continuing students were fitting properly into tertiary education and are gradually becoming conversant with the post senior high school system, adjusting to the demands of tertiary education and also changing learning styles.

This study is consistent with prior studies [42–44] demonstrating that self-efficacy has a strong positive influence on students’ academic performance as students with higher self-efficacy experienced an increase in their CWA when mental health issues were present. Students’ belief in their ability to set challenging goals, deploy effective learning strategies, manage stress, anxiety, and depression, and become resilient positively affects their academic performance. In confirming this finding, [45] reported that self-efficacy not only predicts academic achievement but has also been observed that self-efficacy could improve individual academic performance in China. The greater the level of students’ academic self-efficacy is, the greater their academic commitment, resulting in an advantageous effect on their performance in school [46].

## Limitations and recommendation

This study employed a purely quantitative approach. The lived experiences of respondents concerning mental health and academic performance were not explored. This study did not embrace sufficient variables to evaluate the accompanying coping procedures for mental health issues among the students. Moreover, the Curriculum Weighted Average (CWA) is only a single indicator of academic performance and may not be an accurate reflection of students’ academic improvement. Last, the study was conducted at only one institution. Considering the aforementioned limitations, this study recommends a comparative study across multiple universities to gain comprehensive insight into the research problem. Moreover, future research should regard the adoption of a mixed-method approach for the study of the research problem.

## Conclusion

This study revealed that depression and anxiety are significant risk factors for poor academic performance, and that students' self-efficacy can influence their academic achievement. Compared with males, females are more likely to experience a reduction in their academic performance when challenged with mental health issues. There is an urgent need to recognize the impacts that mental health challenges have on the academic performance of students. The year of study has a significant effect on academic performance when students are challenged with mental health issues. This calls for year-specific mental support and response interventions to propel the holistic sound minds of students while augmenting their academic performance.

## Data Availability

Data is provided within the manuscript file.
